# CircHIPK3 Promotes the Tumorigenesis and Development of Gastric Cancer Through miR-637/AKT1 Pathway

**DOI:** 10.3389/fonc.2021.637761

**Published:** 2021-02-19

**Authors:** Dejun Yang, Zunqi Hu, Yu Zhang, Xin Zhang, Jiapeng Xu, Hongbing Fu, Zhenxin Zhu, Dan Feng, Qingping Cai

**Affiliations:** ^1^ Department of Gastrointestinal Surgery, Changzheng Hospital, Navy Medical University, Shanghai, China; ^2^ Department of Oncology, Changhai Hospital, Navy Medical University, Shanghai, China

**Keywords:** circular RNA, circHIPK3, gastric cancer, miR-637, AKT1 3

## Abstract

Circular RNA is a kind of RNA with a covalently closed loop, which has a complex ability to modulate genes in the process of tumorigenesis and metastasis. Nevertheless, how circular RNA functions in gastric cancer (GC) remains unclear. The effect of circHIPK3 *in vitro* was studied here. Quantitative real-time PCR (qRT-PCR) was employed to found that circHIPK3 markedly increased in GC tissues and cell lines. And low expression of circHIPK3 suppressed the GC cells growing and metabolizing. Then the bioinformatics tool predicted the downstream target of circHIPK3, and it was proved by the dual-luciferase report experiment. According to the results of bioinformatics analysis and experimental data, it was clarified that circHIPK3 acted as a sponge of miR-637, releasing its direct target AKT1. The dual-luciferase assay revealed that mir-637 could bind circHIPK3 and AKT1. qRT-PCR data indicated that overexpression circHIPK3 led to the low level of miR-637 and overexpressed miR-637 would reduce AKT1 level. Finally, we demonstrated that the low expression of miR-637 or overexpression of AKT1 could attenuate the anti-proliferative effects of si-circHIPK3. These results suggest that the circHIPK3/miR-637/AKT1 regulatory pathway may be associated with the oncogene and growth of gastric cancer. In short, a new circular RNA circHIPK3 and its function are identified, and the regulatory pathway of circHIPK3/miR-637/AKT1 in the tumorigenesis and development of gastric cancer is discovered.

## Introduction

### Background

According to recent reports, the number of new gastric cancer patients worldwide was over 10 million in 2018. The mortality of gastric cancer (GC) ranked the second among cancers, leading to the death of 7.83 million people in 2018 (1). *Helicobacter pylori* infection and dietary habits are considered as the main factors that cause gastric cancer (2). The surgery is still the most effective method for the treatment of GC. However, survival in GC patients seems to depend on the stage of the disease with GC (3). Thus, it is extremely important to make an early screening and diagnosis to enhance the effectiveness of treatment. Nowadays, it is possible to detect the disease at its earliest stage by extensive screening programs with the development of new biomarkers, like human epidermal growth factor receptor 2 (HER2) (4). The carcinoma-relevant biomarkers exist as a gradually crucial part in the screening and treatment of cancer (5).

Non-coding RNA (ncRNA), as its name suggests, does not encode a protein. Although these ncRNAs cannot encode proteins, they are indispensable to maintain complex life for an organism (6). NcRNAs are extremely abundant and diverse in different cells. Among them, the ncRNAs that perform relatively general functions are rRNA, tRNA, snRNA, and snoRNA (7). Additionally, lncRNA, circRNA, miRNA, and siRNA also belong to ncRNA as well as exerting specific functions in cellular activities (8, 9). CircRNA (circular RNA) is a part of ncRNA with a covalently closed continuous loop. However, the important role of circRNA for cellular metabolism has not been appreciated until recentness (10). CircRNAs are seen to maintain their relative stability in cells due to the special structure with closed ring (11). Recently, there have been shreds of evidence indicating that circRNAs affect the progression of some diseases by regulating gene expression. For example, Hsiao et al. revealed that circCCDC66 promoted cancer growth and metastasis *via* the regulation of a subset of oncogenes in colon cancer (12). Zhong et al. demonstrated that circRNA-MYLK promoted cell proliferation, migration, HUVEC tube generation, and reorganized cytoskeleton *via* activating VEGFA/VEGFR2 signaling pathway (13).

MicroRNAs (miRNAs) are classified as non-coding RNAs with small size. MiRNAs are rich in cells during different stages of the cell cycle. The main function of miRNA displays critically in the regulation of gene post-transcription (14, 15). The mechanism of regulation is very complex, and there is no simple one-to-one correspondence between miRNAs and target genes (16). Several studies have shown that miRNAs during the initiation and progression of cancer can be involved in the progression of regulation (17, 18). An increasing number of studies indicate that circRNA can act as miRNA sponges in the development of cancer. For example, Yang et al. revealed that circ-ITCH “sponged” miR-17 and miR-224, which up-regulated the expression of downstream target p21 and PTEN, then led to the suppression of the development in bladder cancer (19).

Competitive endogenous RNA (ceRNA) is a transcript with the same miRNA response element (MRE), which can act as a miRNA chelating molecule and compete with miRNA to regulate its target genes, thereby affecting the biological behavior of tumors. circRNAs may act as a reaction element for ceRNA to competitively bind miRNA, thereby regulating the expression level of mRNA which is a target by miRNA. Therefore, circRNA-miRNA-mRNA interaction may be an important mechanism for the initiation and development of human tumors. This hypothesis has been confirmed in previous studies. It was found in cervical cancer that circRNA hsa_circRNA_101996 activates the expression of TPX2 by inhibiting miR-8075, thereby enhancing the proliferation and invasion of cervical cancer. In breast cancer, circRNA_0025202 regulates the sensitivity of Tamoxifen and tumor progression through the regulated miR-182-5p/FOXO3a axis. Hsa_circRNA_103809 regulates cell proliferation and migration in colorectal cancer through the miR-532-3p/FOXO4 axis.

Here, our study revealed that circHIPK3 was aberrantly expressed in GC. The experimental research showed that the knockdown of circHIPK3 inhibited GC cells from proliferating, invading, and migrating *in vitro*. Mechanistically, the results indicated circHIPK3 increased the level of miR-637 downstream target gene AKT1 through “sponging” miR-637, which promoted GC growth. In short, we found that circHIPK3 might serve as a potential biomarker for GC. The experimental results provided new hints for cancer research and treatment.

## Materials and Methods

### Bioinformatics Analysis

To investigate the role of the “miRNA sponge,” Circinteractome (https://circinteractome.nia.nih.gov/) was applied to forecast the feasible miRNAs binding sites of circHIPK3 and respective miRNAs. DIANA-TarBase v8.0 software (http://diana.imis.athena-innovation.gr/DianaTools) was conducted to presume probable targets of miRNA.

### Tissue Samples

Ten pairs of GC and healthy tissues of surgical patients with the clinical profile were from Changzheng Hospital, Navy Medical University, Shanghai, China. After collecting indicated specimens, we immediately put them into liquid nitrogen for snap-frozen and then kept them at −80°C for the following experiments. Our experiments were performed on the premise of the approval of the Human Research Ethics Committee of this hospital. Here, our study got the unanimous consent of all patients with signed informed documents.

### Cell Culture and Transfection

Human gastric cancer cell lines (BGC-823, CRL-5822, SGC-7901, and AGS) and normal gastric epidermal cell lines (GES-1) were maintained in RPMI-1640 medium (BI, Israel) supplied with 10% FBS (BI, Israel) and 1% penicillin/streptomycin (BI, Israel) under 37°C incubator with 5% CO_2_. The siRNA targeting circHIPK3, miR-637 mimic and inhibitor and controls were ordered from Ribobio Biotechnology (Guangzhou, China). The coding sequence of AKT1 was inserted into the pcDNA3.1 construct to obtain an overexpression cassette of AKT1. Use Lipofectamine 2000 (Invitrogen, Carlsbad, CA, USA) to transfect the relative plasmid into GC cells according to the manufacturer’s instructions. After 6 h, change to fresh medium and continue incubating for 24 h. The sequence of the siRNA for the circHIPK3 was 5′- CUACAGGUAUGGCCUCACA-3′ (si-circHIPK3). The sequence of negative control siRNA (si-NC) was 5′- UUCUCCGAACGUGUCACGUTT-3′.

### Construction of Circular RNA Vector

The human coding sequence of circHIPK3 was obtained from Mras genome of SGC-7901 cells. Exon 2 sequence of Mras, 100 bp of upstream and 100 bp of downstream of adjacent sequences were involved. The final recombinant construct of pzw-circHIPK3 was confirmed by sequencing.

### RNA Extraction of Nuclear and Cytoplasmic Fractions

MirVana PARIS™ Kit (AM1556; Ambion, Austin, TX, USA) was used to extract nuclear and cytoplasmic RNA referred to manufacturers described. Then 5 × 10^7^ cells were harvested with low-speed centrifugation to discard medium, and then washed in pre-cold PBS twice, followed by exposed on ice. Then, 400 μl of cell fractionation buffer was added into the cell pellet, and kept on the ice for more than 5 min, followed by centrifuging at 4°C (500 g for 5 min) and harvesting cytoplasmic fraction. Five hundred microliters of Cell Disruption Buffer was mixed with pellets and vortexed to smash nuclei until that cell lysate became homogenized.

### Extraction and Quantitation of RNA

Trizol (Invitrogen, USA) was employed to separate RNA from tissues and cells of patients. Primers for qRT-PCR (quantitative real-time PCR) were ordered from Sangon Biotech (Shanghai, China). GAPDH or U6 was regarded as a reference control. Math Processing Error method was used to assess normalized gene expression.

### Cell Proliferation Assay

Cell Counting Kit-8 (CCK-8, Dojindo Chemical Laboratory, Kumamoto, Japan) was performed to assess cell proliferation. Taken briefly, 1 × 10^3^ cells of each well were cultured in 96-well plates for each group in quadruplicate. Each well was added 10 μl of CCK-8 reagent from 1 day to 5-day post-incubation and then placed at 37°C for 1.5 h. The absorbance value of 450 nm was measured by synergy 2 (Molecular Devices, Bio-Tek, CA, USA).

### Transwell Assay

GC SGC-7901 and AGS cells in 200 μl serum-free medium were ready in the upper trans-well chamber for migration assay or in the upper trans-well chamber with matrigel for invasion assay (8.0 μl pore size; BD Biosciences, Franklin Lakes, USA). Twenty hours later, the cells were fixed in formaldehyde for 10 min and stained with DAPI for 15 min.

### Dual-Luciferase Assay

Then 1 × 10^4^ of SGC-7901 cells for each well were inoculated in 96-well plates. The sponge sequence of circHIPK3, AKT1 3′-UTR comprising assumed miR-637 sponge sites, and respective site-specific mutated seed sequence were ligated into pmirGLO reporter vector (Promega, USA). Co-transfection of miR-637 mimic with expected plasmids at specified concentration referred to website (https://www.promega.com.cn/products/reporter-assays-and-transfection/reporter-vectors-and-cell-lines/pmirglo-dual-luciferase-mirna-target-expression-vector). We harvested total lysates and successively detected them for 48 h at post-transfection.

### Statistical Analysis

All the representative data were indicated as mean ± SD. The differences existing in two or multiple groups were calculated by Student’s t-test. Each group was performed three independent times in triplicate. The obvious difference suggested a *P* value less than 0.05.

## Results

### Analysis of circHIPK3 Expression in GC

**Figure 1:**
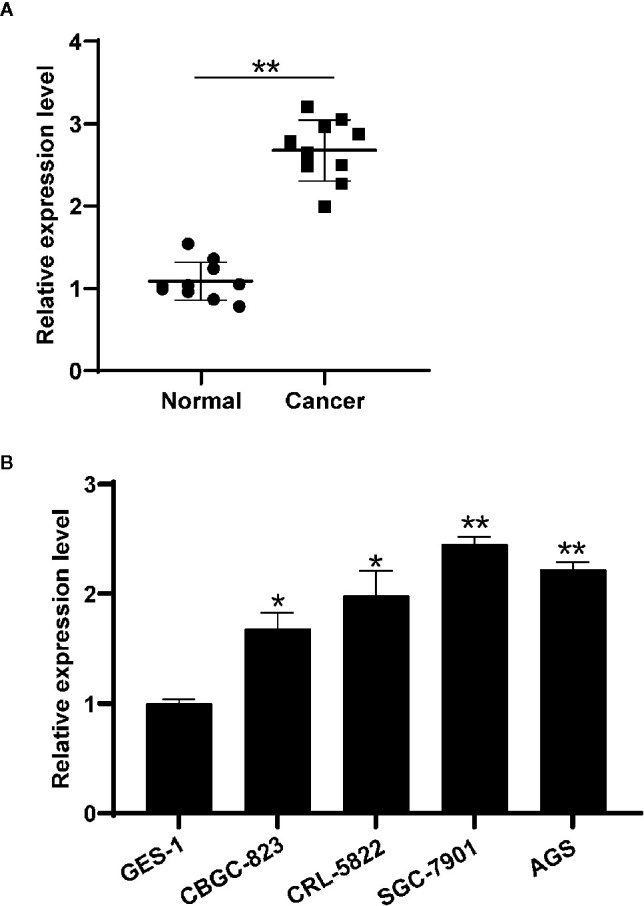
Circ-HIPK3 expressed higher in gastric tissues and cell lines. **(A)** Differential expressions of circHIPK3 in 10 pairs of GC and nearby normal tissues using qRT-PCR, **P < 0.01. **(B)** Differential expression level of circHIPK3 in GC cell lines and normal gastric cell lines (GES-1) using qRT-PCR, *P < 0.05, **P < 0.01.

The differential expressions of circHIPK3 were validated in 10 pairs of GC tissues and nearby normal ones. QRT-PCR data suggested that circHIPK3 expression level was increased in GC relative to that in control normal tissues, revealing circHIPK3 might be a tumor motivator (**Figure 1A**). We then detected circHIPK3 levels in five gastric cell lines. Our data indicated that circHIPK3 level was increased in gastric cancer cell lines (BGC-823, CRL-5822, SGC-7901, and AGS) compared with normal gastric cell line (GES-1) (**Figure 1B**). Collectively, our data revealed that the shape of circHIPK3 presented a circle and an increased level of circHIPK3 was shown in GC cell lines.

### Ablated circHIPK3 Inhibited GC Cell Viability, Migration, and Invasion

**Figure 2:**
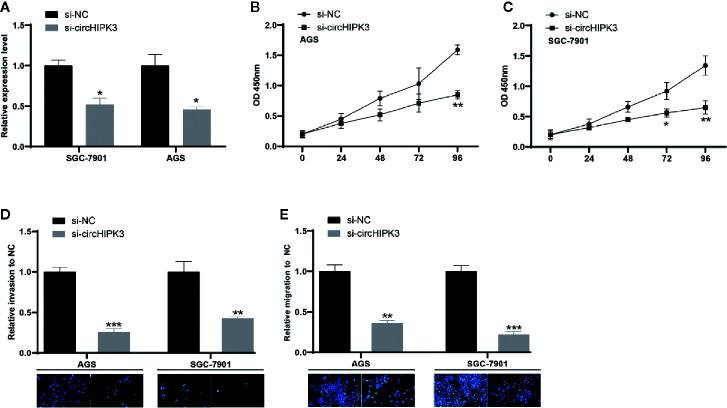
Ablated circHIPK3 inhibited GC cell viability, migration, and invasion **(A)** Relative expression level of circHIPK3 in GC cells transfected with si-circHIPK3 and si-NC, *P < 0.05. **(B, C)** Proliferation ability of **(B)** SGC-7901 and **(C)** AGS cells transfected with si-circHIPK3 and si-NC, *P < 0.05, **P < 0.01. **(D, E)** Cell migration and invasion ability of **(D)** SGC-7901 and **(E)** AGS cells transfected with si-circHIPK3 and si-NC, **P < 0.01, ***P < 0.001.

Then, siRNA was used to reduce the level of circHIPK3 in SGC-7901 and AGS and investigate its “loss of function.” One efficient siRNA targeting back-spliced sequence of circHIPK3 was chosen, and it exhibited strong inhibited effects in SGC-7901 and AGS cells (**Figure 2A**). We herein chose si-circHIPK3 to conduct the following experiments. CCK-8 assay suggested that decreased expression level of circHIPK3 greatly inhibited proliferation of SGC-7901 and AGS cells (**Figures 2B, C**). Additionally, the associated assays revealed the reduced level of circHIPK3 resulted in a decreased ability to migrate and invade (**Figures 2D, E**). Our data suggested that decreased expression level of circHIPK3 prevented GC cells from growing, migrating, and invading.

### CircHIPK3 Played as a Sponge of miR-637 to Induce AKT1

**Figure 3:**
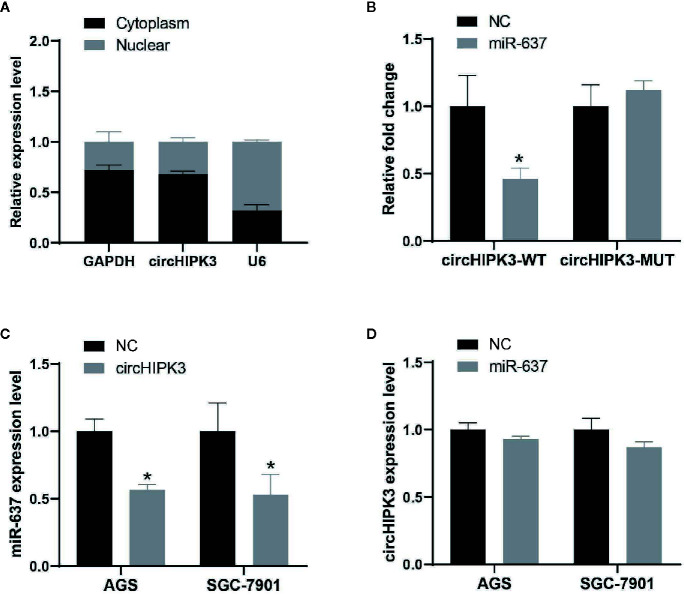
CircHIPK3 acted as a miR-637 sponge in GC **(A)** Subcellular location of circHIPK3 in SGC-7901 cells was detected by qRT-PCR. GAPDH and U6 were used as cytoplasmic and nuclear markers, respectively. **(B)** Luciferase activity of circHIPK3-WT vector was significantly reduced whereas of circHIPK3-MUT vector was not change in SGC-7901 cells co-transfected with miR-637 mimic, *P < 0.05. **(C)** Expression level of miR-637 was downregulated after overexpressing circHIPK3 in AGS and SGC-7901 cells by qRT-PCR analysis, *P < 0.05. **(D)** Expression level of circHIPK3 was not change after overexpressing miR-637 in AGS and SGC-7901 cells by qRT-PCR analysis.

Recently, some researches showed that the main function of circRNA acted as miRNA sponges to target respective miRNAs, followed by regulating gene expression (20). Nucleic and cytoplasmic fraction assays were performed to verify that circHIPK3 was mainly located in cytoplasm (**Figure 3A**). These findings suggested that circHIPK3 may act as a miRNA sponge in GC. Here, bioinformatic analysis revealed that circHIPK3 possessed responsive elements of numerous miRNAs, including miR-637 and DIANA-TarBase v8.0 software revealed that AKT1 was a potential target of miR-637, thus we hypothesized that circHIPK3 played a role in the tumorigenesis and development of GC by regulating miR-637/AKT1 axis.

To further validate the circRNA-miRNA-mRNA regulatory networks, we first evaluated miRNA-binding ability to circHIPK3 by luciferase assay. Our results suggested that the interaction existing in circHIPK3 and miR-637 in SGC-7901 cells resulted in reduced luciferase activity, while no change of luciferase activity was shown in SGC-7901 cells treated with circHIPK3 mutant and miR-637 (**Figure 3B**). Next, we detected the regulation between circHIPK3 and miR-637 in view of interaction existed in circHIPK3 and miR-637. QRT-PCR data indicated that overexpression of circHIPK3 would inhibit miR-637 expression level (**Figure 3C**) whereas heightened miR-637 expression exerted no influence on circHIPK3 expression (**Figure 3D**), indicating that circHIPK3 would display as a regulation executor of miR-637 expression.

**Figure 4:**
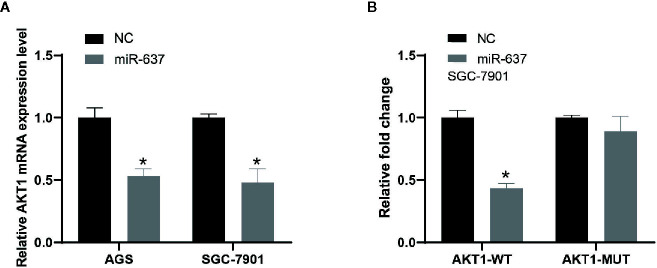
The interaction between AKT1 and miR-637 **(A)** Expression level of AKT1 was downregulated after the overexpression of miR-637 in AGS and SGC-7901 cells by qRT-PCR analysis. **(B)** Luciferase activity was significantly decreased in AKT1-wt group whereas nearly no change in AKT1-mut group after the overexpression of miR-637 in SGC-7901 cell.

Subsequently, the interaction between AKT1 and miR-637 was confirmed. QRT-PCR data indicated that overexpressed miR-637 in AGS and SGC-7901 cells would significantly reduce AKT1 level (**Figure 4A**). The dual-luciferase assay showed that overexpressed miR-637 inhibited the luciferase activity of AKT1 wild type vector, not AKT1 mutant vector (**Figure 4B**).

### CircHIPK3 Enhanced Cells Viability by Regulating miR-637/AKT1 Axis in GC

**Figure 5:**
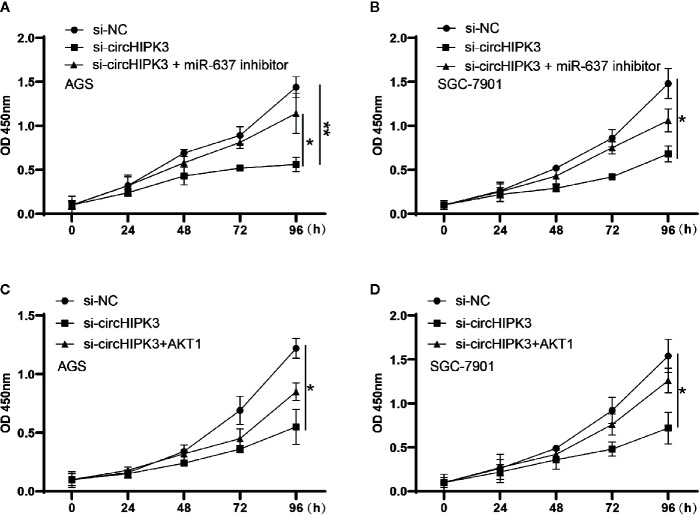
Proliferation measured using CCK-8 assay in cells transfected with si-NC, si-circHIPK3 and the combination of si-circHIPK3 and miR-637 inhibitor or AKT1. The decline in proliferation brought by si-circHIPK3 has been partially offset by **(A, B)** miR-637 low-expression or **(C, D)** AKT1 over-expression in AGS and SGC-7901 cells, *P < 0.05, **P < 0.01.

Finally, we deeply investigated the functional aspects. Proliferation was assessed using a CCK-8 kit in AGS and SGC-7901 cells transfected with si-circHIPK3 and miR-637 inhibitor or AKT1 overexpression cassette. The results revealed that promoted cell viability generated in AGS and SGC-7901 cells with low-expressed circHIPK3 was greatly ablated upon miR-637 low-expression (**Figures 5A, B**) or AKT1 overexpression (**Figures 5C, D**). To sum up, our findings demonstrsted circHIPK3-mediated promotion of GC cell proliferation and migration *via* regulating miR-637/AKT1 axis.

## Discussion

Despite 40 years since its discovery by electron microscope technique, we have not understood circRNA functions until a; breakthrough in the synthesis and sequencing technology of RNA (21, 22). As the development of molecular biology, circRNA function and mechanism has been studied extensively. Numerous studies towards the effects of circRNA on cancer development have been reported before (23). As one of the most widely occurred malignant carcinomas, GC appeared in some areas, such as East Asia and Eastern Europe (24). Several researches have reported that circRNAs exhibited a primary role in GC tumorigenesis and progression. For instance, Li et al. showed that circ-ERBB2 promoted GC cell proliferation by miR-503/CACUL1 signaling and induced cell invasion *via* miR-637/MMP-19 axis (25). Wu et al. demonstrated that circ-DCAF6 promoted GC cell growth and invasion upon inhibiting miR-1231 and miR-1256 level (26). Guan et al. revealed that circ-NOTCH1 strengthened GC cell viability and invasion, but decreased cell apoptosis by hindering the transcriptional activity of miR-637 (27). CircRNAs are important regulatory factors in the developmental progression of GC (28). Up to now, however, there is no report on the roles of circHIPK3 on growth of GC.

Our data revealed that circHIPK3 level increased dramatically in GC tissues and cells after normalized to that in normal ones. From this consideration, we hypothesized that circHIPK3 displayed an elementary role in GC growth. We then performed circHIPK3 knockout experiments using siRNA to elucidate the function of circHIPK3 in GC. Functionally, CCK-8 and Transwell assay both verified that downregulation of circHIPK3 expression largely suppressed GC cell proliferation, migration, and invasion *in vitro*. These experimental results provide initial evidence for our hypothesis.

More circRNA functions are also found by virtue of the progress of molecular biology techniques and increasing research funding. The major function of circRNA is acting as regulators in post-transcriptional regulation, such as circRNA functioning as miRNA sponges in miRNA-mRNA regulatory network (29). To verify our hypothesis and initial results, a dual-luciferase assay was performed. The results have shown circHIPK3 could be bound to miR-637, which circHIPK3 expression level exhibited a negative correlation with miR-637 expression level. Meanwhile, we found that AKT1 might be a downstream target gene of miR-637. AKT1 (one of serine threonine kinase) has been involved in the regulation of cell growth, cell cycle or apoptosis (30). Previous studies have shown that AKT1 promoted mammary epithelial tumor cell migration and invasion *in vivo (* 31). Priolo et al. revealed that activation of AKT1 had links to accumulated aerobic glycolysis metabolites, and acted as an oncogene role in prostate cancer growth (32). In this study, mechanistically, circHIPK3 increased AKT1 *via* inhibiting miR-637, which boosted GC cell viability, migration and invasion. In future studies, we will collect more samples to analyze the expression of circHIPK3, miR-637 and AKT1 in clinical samples, and explore their prognostic value. We will conduct more experiments to identify the functions of circHIPK3, miR-637, and AKT1 in, cell lines and mouse models, and evaluate their characteristics in the development of GC in the following studies.

Taken together, we found that circHIPK3 was overexpressed in GC, mainly located in the cytoplasm, and promoted the proliferation, migration, and invasion of GC cells. In addition, we identified an important role of circHIPK3 in GC cells that induced cell viability, migration, and invasion by regulating miR-637/AKT1 signaling pathway. As present researches described, miR-637 expression level has been regulated by circHIPK3, and AKT1, miR-637 downstream gene, is under the control of miR-637. Our results suggested that the initiation and progression of GC were affected by circRNA-miRNA-mRNA regulatory networks mediated by circHIPK3. In short, we revealed for the first time that the circHIPK3/miR-637/AKT1 regulatory pathway may be related to the tumorigenesis and growth of gastric cancer, our research results provide a potential therapeutic target for GC.

## Data Availability Statement

The original contributions presented in the study are included in the article/supplementary material. Further inquiries can be directed to the corresponding authors.

## Ethics Statement

The studies involving human participants were reviewed and approved by the Human Research Ethics Committee of Changzheng Hospital. The patients/participants provided their written informed consent to participate in this study.

## Author Contributions

Conception and design: DY, ZH, and YZ. Development of methodology: JX, HF, and ZZ. Sample collection: DF. Analysis and interpretation of data: DY and QC. Writing, review, and/or revision of the manuscript: DY, ZH, YZ, and XZ. All authors contributed to the article and approved the submitted version.

## Funding

This work is supported by the National Natural Science Foundation of China (Grant Number 81602420) and the National Natural Science Foundation of China (Grant Number 81772955).

## Conflict of Interest

The authors declare that the research was conducted in the absence of any commercial or financial relationships that could be construed as a potential conflict of interest.
